# Miscellany

**Published:** 1902-11

**Authors:** 


### Miscellany.

TO REMOVE THE ODOR OF IODOFORM FROM THE HANDS.—The odor of iodoform may be removed from the hands by washing with a weak solution of tannic acid, by rubbing freely with chloroform, by bathing them in vinegar, or by ablutions in flaxseed-meal water. —*Journal of Medicine and Science.*

---

THE GREAT CORK-FORESTS OF SPAIN.—The *Scientific American* quotes the following from the *Boston Herald*:

"The cork-forests of Spain cover an area of six hundred and twenty thousand square miles, producing the finest cork in the world. The forests exist in groups, and cover wide belts of territory, those in the region of Catalonia and part of Barcelona being considered the first in importance. Although the cork-forests of Estremadura and Andalusia yield cork of a much quicker growth and possessing some excellent qualities, its consistency is less rigid, and on this account it does not enjoy the high reputation which the cork of Catalonia does.

"In Spain and Portugal, where the cork-tree, or *Quercus suber,* is indigenous, it attains to a height varying from thirty-five to sixty feet, and the trunk to a diameter of thirty to thirty-six inches. This species of the evergreen oak is often heavily caparisoned with wide-spreading branches clothed with ovate oblong evergreen leaves, downy underneath, and the leaves slightly serrated. Annually, between April and May, it produces a flower of yellowish color, succeeded by acorns. Over thirty thousand square miles in Portugal are devoted to the cultivation of cork-trees, though the tree abounds in every part of the country.

"The methods in vogue in barking and harvesting the cork in Spain and Portugal are virtually the same. The barking operation is effected where the tree has acquired sufficient strength to withstand the rough handling it receives during the operation, which takes place when it has attained the fifteenth year of its growth. After the first stripping the tree is left in this juvenescent state to regenerate, subsequent strippings being effected at intervals of not less than three years, and under this process the tree will continue to thrive and bear for upward of one hundred and fifty years."

[The number of square miles the cork-forests cover in Spain and those devoted to the cultivation of cork-trees in Portugal are evidently erroneous, as the area of Spain is but one hundred and ninety-one thousand square miles and that of Portugal but thirty-four thousand four hundred and nineteen.—McClain.]

---

Flies as Carriers of Disease.—For forty years the writer has been struck by the fact that all animals hate flies with an intensity not explained by their suctorial or "biting" powers. Our domestic animals, even our draught animals, expend as much energy in keeping off the pests as they do in our service, or in getting food. Even the most pachydermatous are no exceptions to the rule. If we observe an elephant, we are astonished at his constant watchfulness and exertions in keeping off the common flies whose suctorial powers are so feeble as to be utterly out of proportion to the energy spent. Might not bacteriology have got a hint here of service in discovering the etiology of disease? Did not nature long ago thus find out that flies might be the carriers of disease germs? Science is now advancing rapidly towards the same discovery.

The entomologist of the United States Agricultural Department, Dr. Howard, after long studies, believes that flies are often the active agents in spreading typhoid fever. The bacillus of Eberth has been found in flies, and if Dr. Howard is right, then the profession must educate the public and nurses, and so order hospital-construction and service that flies shall have no access to food.—*American Medicine.*

---

METALLURGICAL SKILL OF CHALDEANS AND BABYLONIANS.—A communication by M. Berthelot in *Comptes Rendus* shows that the Chaldeans and Babylonians were possessed of considerable metallurgical skill. A Babylonian statuette was found to consist of a copper alloy containing 79.5 per cent. of copper, 1.25 per cent. of tin, and 0.8 per cent. of iron. A statuette from Chaldea, estimated to be two thousand two hundred years old, was composed of nearly pure copper containing only a slight proportion of iron, whereas another Chaldean statuette, some four hundred years older, consisted mainly of an alloy of four parts of copper with one part of lead and a trace of sulphur.—*Scientific American.*

---

TREATMENT OF PULMONARY COMPLAINTS WITH AIR FROM LIMESTONE CAVES.—Victims of pulmonary complaints have heretofore been compelled to make inconvenient journeys to the higher altitudes in search of the pure rarefied air which is known to be so beneficial to them, but this is no longer necessary. It has been discovered that the air from limestone caves has all the characteristics of that of the mountains. This discovery has just been made use of in the location of a sanitarium near one of these caves, and the air for the institution is supplied from the underground caverns. This establishment is at Luray, Va., and the system of ventilation is arranged so that each room gets its own supply direct from the cave. The air of these caverns is of a very uniform temperature and remarkably pure and free from all germs and dust particles. In the warmest weather the doors and windows of this institution are kept closed, and a comfortable temperature of 75° F. is maintained, in spite of one of 90° or more encountered outside.—*Scientific American.*

---

HOLDING PATIENTS UNDER THE INFLUENCE OF NITROUS OXIDE GAS.—Dr. F. M. Richardson, of Chicago, gave a very interesting two hours' clinic at the Northwestern Dental School on the use of the Hurd inhaler, for the administration of nitrous oxide gas. He extracted teeth for forty patients without a failure. Much to the astonishment of those present, he held his patients under the influence of the nitrous oxide gas as long as he desired, in several cases for five minutes or more.—*Dental Review.*

---

USE OF BEADS IN RETAINING RUBBER DAM.—At the annual clinic of the Alumni Association of the Northwestern Dental School, Dr. A. B. Freeman, of Chicago, demonstrated by a table clinic the use of beads in retaining the rubber dam. The method is to fasten a bead to the centre of the ligature by passing the ligature through the bead and securing the latter by means of a knot. Then the ligature is passed between two teeth, the bead drawn close to the tooth, and the ligature tied. Then the rubber dam is applied over the bead.—*Dental Review.*

[It seems to the writer that it would be easier and better to apply the ligature and bead after putting on the rubber dam.

I have often used a bit of cotton tied in the centre of the ligature and found it to answer every purpose. This is especially applicable when the rubber dam extends to the bicuspids and saves the use of a clamp. Sandarach varnish applied to the rubber dam about the necks of the teeth will often prevent its slipping off as well as prevent leaking.—MCCLAIN.]

Results obtained by Antityphoid Inoculation.—Professor A. E. Wright, of the Army Medical School at Netley, has published the results obtained by antityphoid inoculation. It is demonstrated, so it is said, that fewer cases and fewer deaths occurred among those inoculated than among those untreated.—*Scientific American.*

---

Gold Inlays.—Dr. L. L. Davis, of Chicago, uses the following method for making a gold inlay: An impression of the cavity is taken in cement. A counter-die of cement of another color is made, using Mennen's borated talcum powder to separate the two. A matrix of pure gold is then swaged and filled by melting into it twenty-two-carat gold scraps. It is then trimmed and set in cement. This sort of inlay was recommended of especial value in buccal cavities in molars and bicuspids where the cavity extends below the free margin of the gum.

Dr. George Evans's method is to use rolled platinized gold to form a matrix for gold inlays in cavities in the front teeth, which are considered too soft to permit of the insertion of gold-foil fillings properly.

His method is, after procuring a good matrix, to build on the surface small particles of gold plate, and fuse together with solder until the contour is nearly reached, then to contour a tip, say to an incisor, with crystal gold and flow solder into that. Very beautiful contours were worked up in that way, and the results show highly satisfactory for that character of work.—*Dental Review.*

---

To remove Vulcanite from between Teeth.—Mount a stiff fine needle in a small handle or broach-holder; sharpen on two sides, and you have a useful little tool.—A. E. H. Leister, *Items of Interest.*

---

Vulcanite Inlays.—For occlusal surfaces inlays of gray or white vulcanizable rubber are readily made at but little cost, the appearance of the finished work being excellent. The cavity is prepared without undercuts, swabbed with vaseline, and an impression taken with mouldable wax. White or gray rubber is packed into the plaster mould and the case vulcanized. Finish up well, polish, and cement to place. Gold- or platinum-foil may be used as a matrix, thus assuring a perfect fit.—*Dental Digest.*

